# Maternal and offspring high-fat diet leads to platelet hyperactivation in male mice offspring

**DOI:** 10.1038/s41598-020-80373-3

**Published:** 2021-01-14

**Authors:** Renato S. Gaspar, Amanda J. Unsworth, Alaa Al-Dibouni, Alexander P. Bye, Tanya Sage, Michelle Stewart, Sara Wells, Roger D. Cox, Jonathan M. Gibbins, Dyan Sellayah, Craig E. Hughes

**Affiliations:** 1 Institute for Cardiovascular and Metabolic Research, School of Biological Sciences, University of Reading - Harborne Building, Reading, RG6 6AS UK; 2 Department of Life Sciences, Faculty of Science and Engineering, Manchester Metropolitan University, John Dalton Building, Manchester, M1 5GD UK; 3 MRC Harwell Institute, Mary Lyon Centre, Harwell Campus, Oxfordshire, OX11 0RD UK; 4 MRC Harwell Institute, Genetics of type 2 diabetes, Mammalian Genetics Unit, Harwell Campus, Oxfordshire, OX11 0RD UK

**Keywords:** Platelets, Metabolic syndrome, Platelets, Metabolic diseases

## Abstract

Maternal over-nutrition increases the risk of diabetes and cardiovascular events in offspring. While prominent effects on cardiovascular health are observed, the impact on platelet physiology has not been studied. Here, we examined whether maternal high-fat diet (HF) ingestion affects the platelet function in lean and obese offspring. C57BL6/N mice dams were given a HF or control (C) diet for 8 weeks before and during pregnancy. Male and female offspring received C or HF diets for 26 weeks. Experimental groups were: C/C, dam and offspring fed standard laboratory diet; C/HF dam fed standard laboratory diet and offspring fed HF diet; HF/C and HF/HF. Phenotypic and metabolic tests were performed and blood collected for platelet studies. Compared to C/C, offspring HF groups were obese, with fat accumulation, hyperglycaemia and insulin resistance. Female offspring did not present platelet hyperactivity, hence we focused on male offspring. Platelets from HF/HF mice were larger, hyperactive and presented oxidative stress when compared to C/C. Maternal and offspring HF diet results in platelet hyperactivation in male mouse offspring, suggesting a novel ‘double-hit’ effect.

## Introduction

The concept that the in utero environment impacts long-term metabolic health was first introduced in the late 1980s by Prof David Barker at the University of Southampton^1^. Analysing data from large cohort studies, he noted that infants of low birth weight (a proxy for in utero malnutrition), had increased risk of developing cardiovascular disease and dying of acute thrombotic events (such as stroke and myocardial infarction) in adulthood^2,3^. This concept of developmental programming of cardiovascular disease has since been expanded to include maternal over-nutrition, which is more reflective of Western lifestyles. Both epidemiological and animal studies have established unequivocally that maternal diabetes, obesity or maternal high-fat (HF) diet during pregnancy is associated with increased susceptibility to cardiovascular disease in offspring (reviewed by Williams et al.^4^), although the precise mechanisms underlying this observation remain to be established.

A key component of cardiovascular disease pathophysiology is platelet reactivity, which has been somewhat neglected in the developmental programming field. Platelets contribute to the initiation and development of atherosclerosis through their interaction with activated endothelium and inflammatory cells^5–7^. From a clinical perspective however, their major role is at the later stages of disease when platelets can form unwanted thrombi on atherosclerotic plaques, or when unstable plaques rupture. These acute thrombotic events cause stenosis or occlusion of vessels supplying the heart or brain resulting in myocardial infarction (MI) or stroke respectively (for review see^8^). To this end, a recent cohort study has documented that increased platelet activation in participants before the onset of cardiovascular disease, was a major risk factor and predictor for myocardial infarction and stroke events later in life^9^.

Various components of the cardiovascular system of offspring appear to be negatively affected by in utero over-nutrition^10–13^. Studies in sheep and rodents documented that maternal diet-induced obesity during pregnancy led to endothelial dysfunction, cardiac remodelling, cardiac inflammation and fibrosis, cardiac hypertrophy and impaired cardiac contractility in offspring^10–13^. No measurement of platelet function was reported in those studies. The concept that cardiovascular disease in later life might be programmed during development by maternal over-nutrition is particularly alarming given that the UK has the highest rate of obesity amongst women of reproductive age in Europe^14^. Likewise, maternal diabetes during pregnancy increased early onset of cardiovascular disease in offspring^15^.

Despite strong evidence for developmental programming of cardiovascular disease and the key role of platelets on cardiovascular disease pathophysiology, there is currently no literature assessing the effects of maternal metabolic dysfunction on the platelet reactivity of the offspring. Using a mouse model, we set out to examine the effects of maternal and offspring HF diet ingestion on the platelet function of 30-week-old male offspring mice. Our data showed an overall effect of increased platelet reactivity on offspring born to HF dams and a worsened phenotype if offspring were also fed HF. This suggests that there is a ‘double-hit’ effect in which metabolic dysfunction in both mother and offspring potentiate platelet reactivity. This is a novel study to describe the impacts of maternal HF ingestion on the platelet reactivity of the offspring and offers a possible pathophysiological mechanism that may explain previous reports showing increased cardiovascular events related to maternal metabolic dysfunction.

## Results

### Maternal high-fat diet programmed phenotypic and metabolic parameters differently in lean and obese offspring

Several aspects of the metabolic phenotype as well as serum biochemistry were assessed in 30 weeks old male mice (Table 1). Both offspring HF groups presented significant increases in body weight, fat mass, total cholesterol, fasting glucose, TyG Index which is a surrogate for insulin resistance, as well as HDL and LDL levels when compared to offspring C groups. Interestingly, HF/C animals were lighter and had increased lean mass as percentage of body weight when compared to C/C. HF/HF, but not C/HF, showed increased serum levels of triglycerides and free fatty acids (FFAs) when compared to C/C group, suggesting that a combination of maternal and offspring HF diet is required to raise serum triglyceride and FFA levels. In spite of this, however, for both triglycerides and FFAs there was a significant overall offspring diet effect but not maternal diet effect.

**Table 1:** The effects of maternal and male offspring high-fat diet in metabolic parameters of offspring.

	C/C n = 17	C/HF n = 10	HF/C n = 16	HF/HF n = 13	MD	OD	MD × OD
					*p-values*		
Final weight (g)	35.51 ± 1.043	**51.21 ± 1.174**^**a,c**^	**32.17 ± 1.030**^**a,b,d**^	**49.96 ± 0.675**^**a,c**^	**0.0312**	** < 0.0001**	0.3205
Lean weight (%)	72.34 ± 1.878	**57.58 ± 1.279**^**a,c**^	**79.07 ± 1.84**^**a,b,d**^	**56.98 ± 1.273**^**a,c**^	0.0808	** < 0.0001**	**0.0380**
Fat mass (%)	28.62 ± 2.773	**48.01 ± 1.182**^**a**^	**26.55 ± 1.697**^**d**^	**48.02 ± 1.203**^**a,c**^	0.6296	** < 0.0001**	0.6253
Total Cholesterol (mmol/L)	3.991 ± 0.250	**6.809 ± 0.547**^**a**^	**3.653 ± 0.147**^**d**^	**6.928 ± 0.240**^**a,c**^	0.7154	** < 0.0001**	0.4472
HDL (mmol/L)	2.439 ± 0.157	**3.839 ± 0.260**^**a**^	**2.254 ± 0.097**^**d**^	**3.858 ± 0.092**^**a,c**^	0.6031	** < 0.0001**	0.5217
LDL (mmol/L)	1.12 ± 0.059	**1.97 ± 0.188**^**a**^	**0.8256 ± 0.071**^**d**^	**1.768 ± 0.124**^**a,c**^	**0.0240**	** < 0.0001**	0.6650
Triglycerides (mmol/L)	1.22 ± 0.069	1.418 ± 0.105	1.283 ± 0.105	**1.661 ± 0.169**^**a**^	0.1940	**0.0166**	0.4432
Free fatty acids (mmol/L)	0.6994 ± 0.034	0.881 ± 0.039	0.7888 ± 0.049	**0.9338 ± 0.068**^**a**^	0.1657	**0.0021**	0.7197
Fasting glucose (mmol/L)	7.435 ± 0.330	**11.21 ± 0.371**^**a**^	**6.613 ± 0.256**^**d**^	**9.717 ± 0.589**^**a,c**^	**0.0049**	** < 0.0001**	0.3985
TyG index	8.842 ± 0.080	**9.419 ± 0.087**^**a**^	**8.756 ± 0.087**^**d**^	**9.378 ± 0.114**^**a,c**^	0.5063	** < 0.0001**	0.8101

Data presented as mean ± SEM. N = 10–17 animals from 7–9 litters per group. Groups were analysed by Two-way ANOVA and Tukey’s post-test for multiple comparisons.

Bold values depict statistical differences. The interaction between the two variables denotes that the effect of one variable is dependent on the other.

*C/C* dam and offspring fed standard laboratory diet; *C/HF* dam fed standard laboratory diet and offspring fed high-fat diet; *HF/C* dam fed high-fat diet and offspring fed standard laboratory diet; *HF/HF* dam and offspring fed high fat diet, *MD* maternal diet, *OD* offspring diet, *MDxOD* interaction between MD and OD, *HDL* high-density lipoprotein, *LDL* low-density lipoprotein.

^a^p < 0.05 *vs* C/C.

^b^p < 0.05 *vs* C/HF.

^c^p < 0.05 *vs* HF/C.

^d^p < 0.05 *vs* HF/HF.

**Figure 1:**
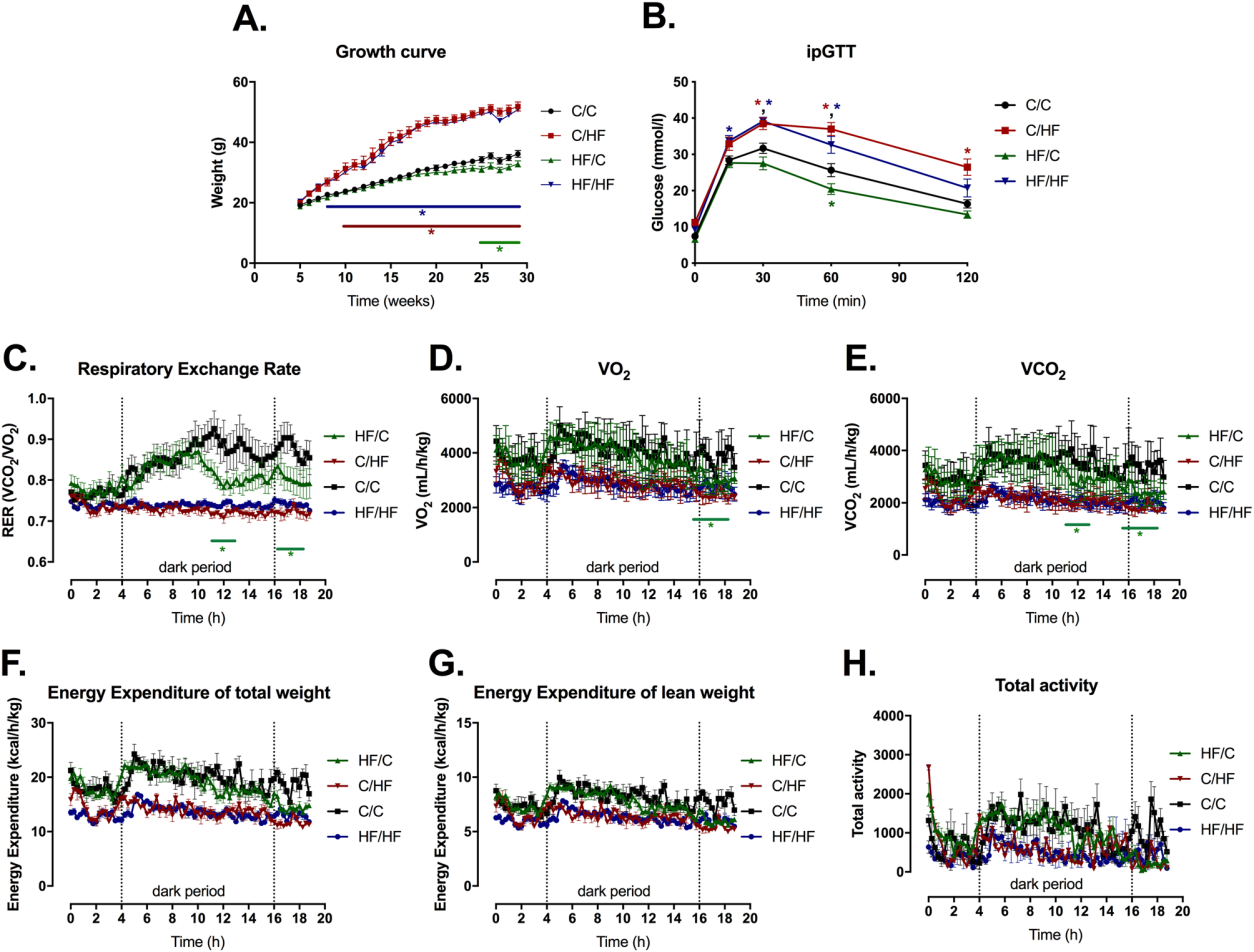
Metabolic effects of maternal obesity depend on male offspring diet. (**A**) Growth curve with weekly weight measures. (**B**) Intraperitoneal glucose tolerance test (ipGTT) with glucose measured at 0, 15, 30, 60 and 120 min after glucose injection. (**C**) Respiratory exchange rate (RER), (**D**) maximum rate of oxygen consumption (VO_2_), (**E**) maximum rate of carbon dioxide consumption (VCO_2_), (**F**) energy expenditure of total weight, (**G**) energy expenditure of lean weight and (H) total ambulatory activity were measured in individual mice placed in a metabolic cage for 20 h. C/C, dam and offspring fed standard laboratory diet; C/HF dam fed standard laboratory diet and offspring fed high-fat diet; HF/C dam fed high-fat diet and offspring fed standard laboratory diet; HF/HF dam and offspring fed high-fat diet. For (**A**) and (**B**), n = 10–17 mice from 7–9 litters per group. For (**C**–**H**), n = 4 animals from 3 litters for C/C, n = 3 animals from 3 litters for C/HF, n = 8 animals from 4 litters for HF/C and n = 8 animals from 4 litters for HF/HF. Data on graphs show mean ± SEM. Data analysed by repeated measures two-way ANOVA with Tukey’s multiple comparisons test. *p < 0.05 and colour of stars indicate significance of the group against C/C.

All groups were also assessed for their body weight growth, glucose homeostasis and indirect calorimetry to further characterize the model (Fig. 1). HF/HF animals were consistently heavier than C/C starting at 7 weeks (p < 0.01), whereas C/HF started at 8 weeks of age (Fig. 1A). Of note, HF/C mice were lighter than C/C over the last 4 weeks of the study (Fig. 1A). IpGTT was performed and evidenced that both offspring HF groups were glucose intolerant, whereas HF/C showed improved glucose homeostasis compared to C/C (Fig. 1B). Interestingly, female offspring C/HF and HF/HF groups displayed similar metabolic dysfunction as the male offspring (Supplementary Fig. S3). Overall, both C/HF and HF/HF presented severe metabolic dysfunction, while male HF/C were lighter and had improved ipGTT when compared to C/C.

Indirect calorimetry was measured from individual animals placed in a metabolic cage. Due to restricted cage availability, sample size was reduced. Offspring HF groups showed a lower RER coupled with decreased energy expenditure, suggesting increased burning of fat and diminished metabolic activity respectively (Fig. 1C–G), which would contribute to the other metabolic dysfunctions present in these animals. Surprisingly, HF/C showed a slightly lower RER compared to C/C, not as pronounced as offspring HF groups. Total ambulatory activity was not changed across groups, likely due to the high variability of the assay (Fig. 1H). Future experiments with increased sample size, will be required to strengthen the interpretation of these data. In summary, maternal HF programmed the offspring differently: if the offspring was given a high-fat diet, they presented more severe metabolic dysfunctions in terms of triglycerides and FFAs levels; if given standard laboratory diet, offspring were lighter and had improved glucose homeostasis.

### Maternal high-fat diet programmes brown adipose tissue of offspring differently, depending on the offspring diet

**Figure 2:**
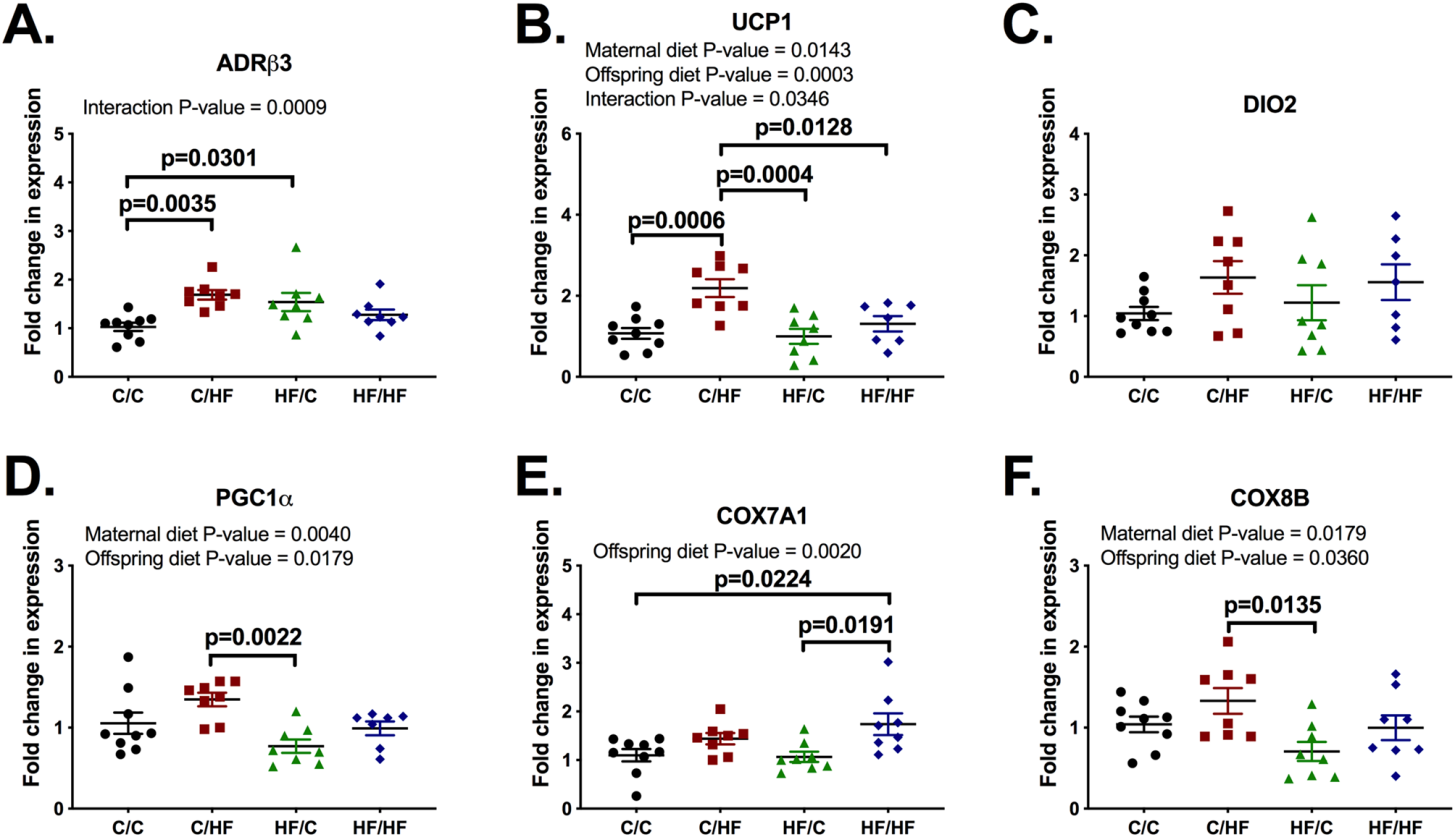
The mRNA levels of brown adipose tissue are affected by maternal and offspring high-fat diet in males. ADRβ3 (**A**), UCP1 (**B**), DIO2 (**C**), PGC1α (**D**), COX7A1 (**E**) and COX8B (**F**) mRNA were quantified in brown adipose tissue of 30-week old male offspring and fold change was determined using the 2^−∆∆^Ct method. C/C, dam and offspring fed standard laboratory diet; C/HF dam fed standard laboratory diet and offspring fed high-fat diet; HF/C dam fed high-fat diet and offspring fed standard laboratory diet; HF/HF dam and offspring fed high-fat diet. Data on graphs show mean ± SEM as well as individual points. n = 7–9 mice from 4 to 6 litters per group. Data analysed by two-way ANOVA with Tukey’s multiple comparisons test. P-values as well as the overall effects of maternal and offspring diet are reported where significant.

To address some of the metabolic dysfunctions observed in maternal and/or offspring HF groups, we quantified mRNA levels of genes associated with thermogenesis (Fig. 2A–C) and mitochondrial function (Fig. 2D–F) in BAT of 30 weeks old male offspring. HF/C and C/HF had increased mRNA levels of adrenergic receptor β3 (ADRβ3) when compared to C/C, whereas only C/HF displayed increased mRNA levels of mitochondrial uncoupling protein 1 (UCP1). Moreover, only HF/HF mice presented increased mRNA levels of cytochrome c oxidase subunit 7A1 (COX7A1) when compared to C/C. Increased ADRβ3 and UCP1 mRNA in C/HF may indicate an adaptive response to HF diet ingestion, whereas the lack of increase in HF/HF suggests maternal obesity aggravates metabolic effects of HF diet in offspring. Likewise, increased ADRβ3 mRNA in BAT of HF/C mice indicates higher adrenergic output, which is in line with decreased weight and improved glucose homeostasis of HF/C mice. Altogether, these data suggest improved metabolic function in HF/C mice may be a consequence of increased adrenergic signalling, while maternal and offspring HF diet disrupted adaptive responses in BAT of HF/HF, thus potentiating metabolic dysfunction.

### Maternal and offspring high-fat diet ingestion led to increased platelet volume

Increased mean platelet volume (MPV) is correlated with cardiovascular events in males^16^, therefore full blood count was performed (Table 2). There was a 30% decrease in total leukocytes on the HF/C group compared to C/C, which suggests leukocytes could be programmed by maternal HF diet ingestion, although there was no overall effect (two-way ANOVA, p = 0.06). HF/HF animals had a 0.7 fL increase in MPV when compared to C/C, while there was no difference when compared to C/HF. Indeed, MPV was significantly increased by maternal (two-way ANOVA, maternal diet effect p = 0.01) and offspring (p = 0.02) HF diet. Since there was no interaction between maternal and offspring HF diet, this suggests a double-hit effect in which both maternal and offspring diet are needed to increase platelet size.

**Table 2:** Maternal and offspring high-fat diet leads to increased platelet volume of male offspring.

	C/C n = 17	C/HF n = 10	HF/C n = 16	HF/HF n = 13	MD	OD	MD × OD
					*P-values*		
**Red blood cells**							
Hematocrit (L/L)	0.483 ± 0.004	0.478 ± 0.013	0.500 ± 0.005	0.492 ± 0.013	0.089	0.494	0.892
RBC (× 10^6^ cells/µL)	9.382 ± 0.122	9.462 ± 0.211	9.715 ± 0.101	9.612 ± 0.303	0.219	0.953	0.639
Haemoglobin (g/dL)	13.81 ± 0.130	12.78 ± 0.641	13.69 ± 0.181	13.41 ± 0.397	0.450	0.056	0.266
MCV (fL)	51.05 ± 0.281	51.24 ± 0.470	51.39 ± 0.402	51.12 ± 0.340	0.781	0.923	0.541
MCH (pg)	14.38 ± 0.165	**13.14 ± 0.581**^**a**^	13.95 ± 0.233	14.08 ± 0.212	0.389	0.064	**0.022**
MCHC (g/dL)	28.19 ± 0.274	**25.94 ± 0.867**^**a**^	27.35 ± 0.286	27.42 ± 0.289	0.462	**0.013**	**0.009**
CHCM (g/dL)	26.29 ± 0.205	**25.12 ± 0.620**^**a**^	25.73 ± 0.236	25.63 ± 0.184	0.931	**0.039**	0.080
RDW (%)	13.57 ± 0.271	**15.29 ± 0.849**^**a,c**^	**13.08 ± 0.159**^**b**^	13.97 ± 0.130	**0.019**	**0.001**	0.269
**White blood cells**							
Leukocytes (× 10^3^ cells/µL)	6.099 ± 0.615	5.297 ± 0.502	**4.322 ± 0.376**^**a**^	5.189 ± 0.174	0.068	0.949	0.105
Neutrophils (× 10^3^ cells/µL)	0.590 ± 0.061	0.524 ± 0.059	0.534 ± 0.055	0.503 ± 0.023	0.477	0.367	0.736
Neutrophils (%)	12.59 ± 1.060	9.456 ± 1.143	11.56 ± 0.785	**8.946 ± 0.499**^**a**^	0.398	**0.002**	0.774
Lymphocytes (× 10^3^ cells/µL)	4.086 ± 0.267	4.256 ± 0.383	3.673 ± 0.363	4.853 ± 0.310	0.784	**0.049**	0.137
Lymphocytes (%)	80.19 ± 1.371	83.63 ± 1.674	82.09 ± 0.937	84.00 ± 0.954	0.358	**0.034**	0.532
Monocytes (× 10^3^ cells/µL)	0.19 ± 0.017	0.167 ± 0.028	**0.1013 ± 0.011**^**a,d**^	**0.19 ± 0.016**^**c**^	0.078	0.078	**0.003**
Monocytes (%)	3.194 ± 0.209	3.18 ± 0.486	2.294 ± 0.230	3.354 ± 0.309	0.224	0.082	0.075
Eosinophils (× 10^3^ cells/µL)	0.103 ± 0.013	0.095 ± 0.017	0.138 ± 0.028	0.096 ± 0.010	0.372	0.220	0.402
Eosinophils (%)	1.813 ± 0.265	1.82 ± 0.291	2.619 ± 0.376	1.754 ± 0.253	0.250	0.184	0.177
**Platelets**							
Platelet count (× 10^3^ cells/µL)	1530 ± 82.88	1366 ± 121.5	1468 ± 116.5	1308 ± 73.33	0.560	0.120	0.986
MPV (fL)	8.112 ± 0.172	8.46 ± 0.117	8.513 ± 0.111	**8.8 ± 0.105**^**a**^	**0.011**	**0.029**	0.831
PDW (%)	48.04 ± 0.899	49.71 ± 0.941	**48.00 ± 0.611**^**d**^	**51.88 ± 0.917**^**a,c**^	0.231	**0.002**	0.216
PCT (%)	1.245 ± 0.075	1.158 ± 0.106	1.253 ± 0.100	1.151 ± 0.067	0.999	0.301	0.936

Data presented as mean ± SEM. N = 10–17 animals from 7–9 litters per group. Groups were analyzed by Two-way ANOVA and Tukey’s post-test for multiple comparisons. Bold values depict statistical differences. The interaction between the two variables denotes that the effect of one variable is dependent on the other.

*C/C* dam and offspring fed standard laboratory diet; *C/HF* dam fed standard laboratory diet and offspring fed high-fat diet; *HF/C* dam fed high-fat diet and offspring fed standard laboratory diet; *HF/HF* dam and offspring fed high fat diet, *MD* maternal diet, *OD* offspring diet, *MDxOD* interaction between MD and OD, *RBC* red blood cells, *MCV* mean corpuscular volume, *MCH* mean corpuscular haemoglobin, *MCHC* mean corpuscular haemoglobin concentration, *CHCM* cell haemoglobin concentration, *RDW* red cell distribution width, *MPV* mean platelet volume, *PDW* platelet distribution width, *PCT* plateletcrit.

^a^p < 0.05 *vs* C/C.

^b^p < 0.05 *vs* C/HF.

^c^p < 0.05 *vs* HF/C.

^d^p < 0.05 *vs* HF/HF.

### Maternal and offspring high-fat diet ingestion increases offspring platelet adhesion and spreading to collagen

**Figure 3:**
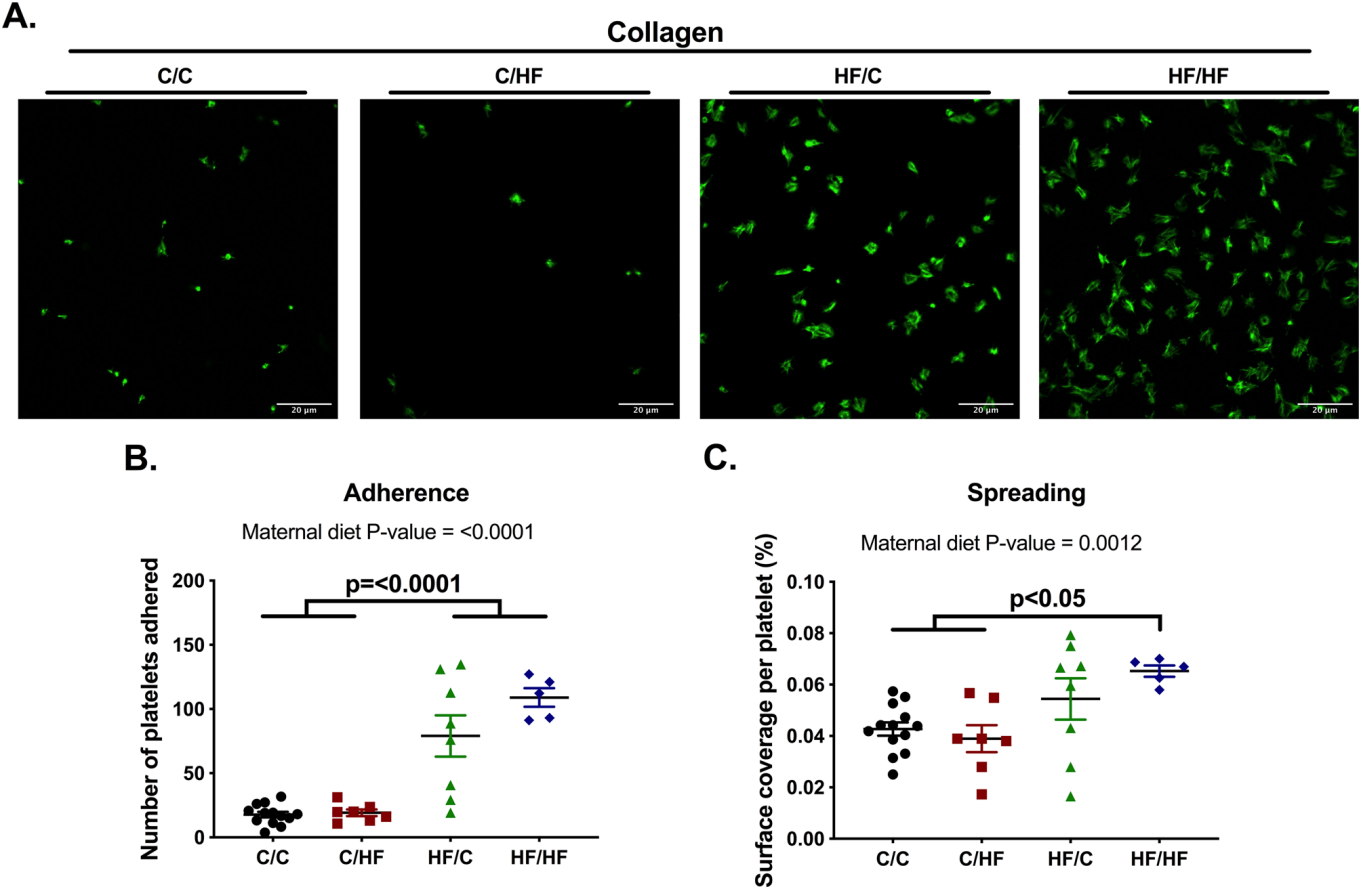
Maternal high-fat diet increased platelet spreading over collagen in male offspring. WP (2 × 10^7^ platelets/mL) was left to adhere to coverslips coated with collagen and platelets fluorescently labelled for visualization. (**A**) Representative images of platelet spreading of each group. (**B**) Quantification of platelet adherence as number of platelets per field. (**C**) Platelet spreading as the total fluorescence of the field divided by the number of platelets. C/C, dam and offspring fed standard laboratory diet; C/HF dam fed standard laboratory diet and offspring fed high-fat diet; HF/C dam fed high-fat diet and offspring fed standard laboratory diet; HF/HF dam and offspring fed high-fat diet. N = 5–13 mice from 4 to 6 litters per group. Graphs show mean ± SEM as well as individual values. Data analysed by two-way ANOVA with Tukey’s multiple comparisons test. P-values as well as the overall effects of maternal and offspring diet are reported where significant.

Upon vascular injury, platelets are exposed and adhere to collagen. Therefore, we assessed the ability of platelets to adhere and spread to immobilized collagen and undergo cytoskeletal reorganisation, referred to as spreading (Fig. 3). When compared to C/C and C/HF, platelets from HF/HF group exhibited a 9 and 1.5 fold increase in platelet adherence and spreading, respectively. This effect was not seen in C/HF, whilst there was an increase in adherence in the HF/C group. The overall effect of maternal high-fat diet ingestion led to increased adhesion (two-way ANOVA, p < 0.0001) and spreading (p = 0.0012) independent of offspring diet, since there was no interaction between maternal and offspring diet, supporting a ‘double-hit’ effect of maternal and offspring HF diet.

### ‘Double-hit’ effect of maternal and offspring high-fat diet ingestion results in platelet hyperactivity and decreased surface expression levels of collagen receptors

**Figure 4:**
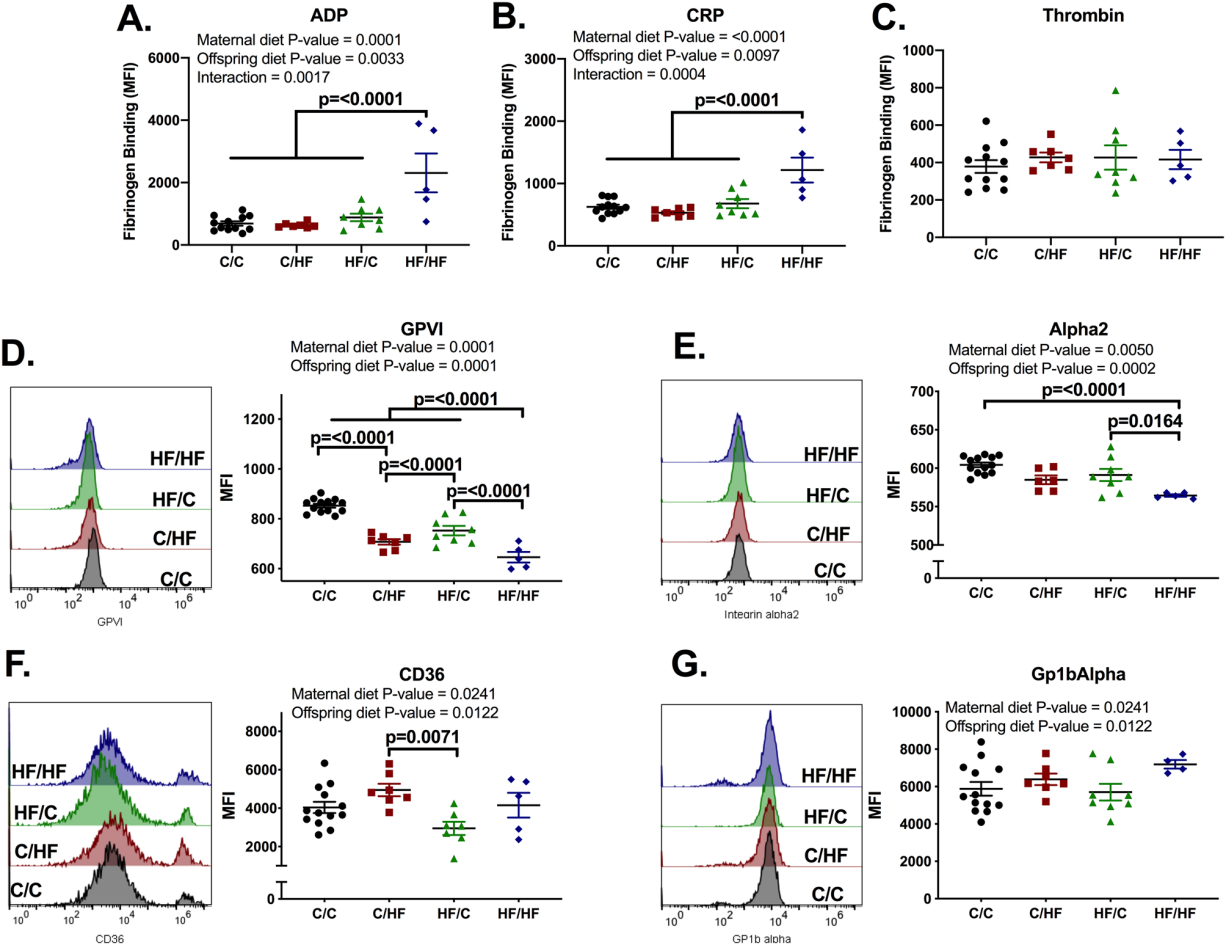
Maternal and offspring high-fat diet ingestion resulted in platelet hyperactivation of male offspring. Platelet-rich plasma (PRP) was stimulated with 10 μM ADP (**A**), 3 μg/mL CRP (**B**) or 0.1 U/mL Thrombin (**C**) and FITC-conjugated fibrinogen binding measured through flow cytometry. Resting PRP was incubated with antibodies for GPVI (D), α2 integrin (**E**), CD36 (**F**) and Gp1bα (**G**) receptors and measured using flow cytometry. C/C, dam and offspring fed standard laboratory diet; C/HF dam fed standard laboratory diet and offspring fed high-fat diet; HF/C dam fed high-fat diet and offspring fed standard laboratory diet; HF/HF dam and offspring fed high-fat diet. N = 5–13 mice from 4 to 6 litters per group. Graphs show mean ± SEM as well as individual values. Data analysed by two-way ANOVA with Tukey’s multiple comparisons test. P-values as well as the overall effects of maternal and offspring diet are reported where significant.

Next, we assessed platelet activation by measuring fibrinogen binding to platelet integrin αIIbβ3 using FITC-conjugated fibrinogen (Fig. 4). PRP was isolated from whole blood and stimulated with different concentrations of ADP, CRP (a GPVI collagen receptor agonist) or thrombin. Platelets of HF/HF animals bound to fibrinogen 3 × more than C/C when stimulated with ADP and 2 × more when stimulated with CRP (Fig. 4A,B), however, for ADP there was an interaction between maternal and offspring diet. Since only platelets from HF/HF mice were hyperactive, this indicates a ‘double-hit’ effect of maternal and offspring HF diet culminating in increased platelet activation. Interestingly, there was no increase when platelets were stimulated with thrombin (Fig. 4C), suggesting that rises in fibrinogen binding are specific to some agonists and are unlikely to be due to increased MPV in HF/HF. The abovementioned effects were similar when different doses of agonists were used or when platelets were resting (Supplementary Fig. S4). Of note, platelet activation was not correlated with circulating lipids (Supplementary Fig. S5). Likewise, platelet hyperactivation was not observed in female offspring, suggesting a sex-specific effect (Supplementary Fig. S3), which led us to focus on the male offspring.

Surface expression levels of key platelet receptors were measured through flow cytometry. Both offspring HF groups showed decreased levels of GPVI and integrin α2 compared to C/C (Fig. 4D,E), indicating increased spreading on collagen and CRP-induced activation observed in HF/HF platelets was not due to upregulated GPVI surface expression. Interestingly, maternal obesity resulted in decreased expression levels of collagen receptors, suggesting possible epigenetic regulation. There were no differences between groups on surface levels of oxidized LDL receptor CD36 or von Willebrand factor receptor CD42b (Fig. 4F,G), despite overall effects of maternal and offspring diets.

### Platelets from HF/HF mice presented increased oxidative stress

**Figure 5:**
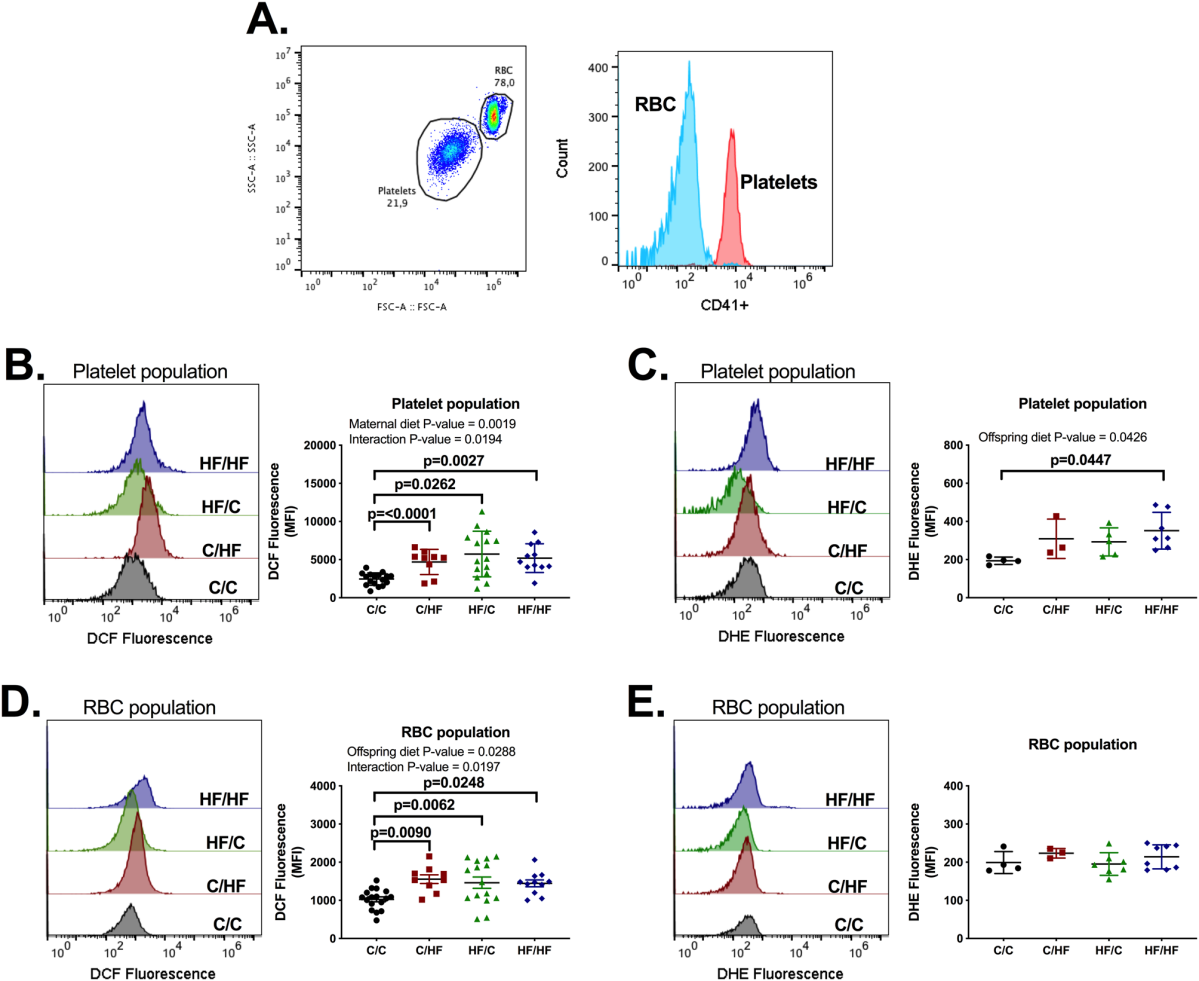
Maternal or offspring high-fat diet induced increased oxidative stress in whole blood of male offspring. Whole blood was diluted 25 times with Tyrodes-HEPES buffer and platelets population labelled with CD41 + antibody for 30 min (**A**). Whole blood was incubated with 20 µM DCFDA or 10 µM DHE and fluorescence determined for platelet (**B**,**C**) and RBC (**D**,**E**) populations using a flow cytometer. C/C, dam and offspring fed standard laboratory diet; C/HF dam fed standard laboratory diet and offspring fed high-fat diet; HF/C dam fed high-fat diet and offspring fed standard laboratory diet; HF/HF dam and offspring fed high-fat diet. For (**B**) and (**D**), n = 10–17 mice from 7–9 litters per group. For (**C**) and (**E**), n = 4 animals from 3 litters for C/C, n = 3 animals from 3 litters for C/HF, n = 5 animals from 4 litters for HF/C and n = 7 animals from 4 litters for HF/HF. Graphs show mean ± SEM as well as individual values. Data analysed by two-way ANOVA with Tukey’s multiple comparisons test. The overall effects of maternal and offspring diet are reported where significant.

ROS generation was measured in whole blood (Fig. 5A) to test whether oxidative stress, often associated with metabolic dysfunction, could contribute to the observed platelet hyperactivity seen in HF/HF group. Both maternal and offspring HF groups presented increased oxidative stress measured using DCF in platelets and red blood cells (RBC) (Fig. 5B,D). However, only platelets from HF/HF mice displayed increased DHE fluorescence when compared to C/C, suggesting only this group had increased generation of ROS (Fig. 5C,E). Additionally, there was no difference between C/HF and HF/HF while there was an overall effect for offspring diet for DHE measured in platelets (Fig. 5C). In spite of this, the reduced sample size for C/HF and HF/C (n = 3–4) limits interpretation. Therefore, the increase in ROS of platelets from HF/HF mice may contribute to the increase platelet functional herein observed.

## Discussion

This study describes the impact of maternal obesity during pregnancy in mice on the platelet activity of offspring. We show that HF animals born to HF dams exhibited metabolic dysfunction, with higher serum levels of triglycerides, and developed elevated body weight earlier than HF offspring whose mothers were lean (p < 0.01 at week 7). HF/HF mice also exhibited disrupted adaptive responses in BAT, which corroborates the metabolic dysfunctions observed in these mice. Maternal and offspring obesity led to a ‘double-hit’ effect, characterized by increased spreading over collagen, decreased surface expression of collagen receptors and increased oxidative stress. Platelets from HF/HF animals were larger and hyperactive to several agonists, suggesting multiple mechanisms leading to increased platelet activation. Therefore, we suggest a novel ‘double-hit’ effect of maternal and offspring high-fat diet ingestion that causes platelet hyperactivation in the offspring.

We first assessed metabolic and phenotypic parameters to characterize our model. Surprisingly, we observed that offspring of dams fed a high-fat diet exhibited reduced body weight from 18 weeks of age when maintained on a standard laboratory diet (HF/C) compared the their counterparts from dams who were maintained on a standard laboratory diet during pregnancy (C/C). We observed that HF/C mice had increased mRNA levels of ADRβ3, suggesting increased adrenergic signalling and BAT activation^17^. The majority of studies assessing effects of maternal obesity on offspring have used offspring at a younger age and showed increased or unchanged body weight and adiposity in standard laboratory diet-fed male offspring born to obese dams^18,19^. Similar to our data, Blackmore et al.^20^ showed male mice born to obese dams to have a trend towards lighter body weight and improved metabolic parameters at 12 and 8 weeks of age, respectively. This group has also described an increased sympathetic activity in the heart of these mice, compared to mice born to lean dams^20^. It is possible that maternal obesity may program the offspring to adapt to a fat-rich environment through higher adrenergic discharge, potentially explaining the increased tendency to burn fat. This may represent an adaptive response to perceived nutritional stress such as those termed ‘predictive adaptive responses’^21^. This is in line with our data showing reduced RER, decreases body weight and improved glucose homeostasis in HF/C offspring. It is not clear, however, why these potential adaptive changes occur at a delayed point during the life-course of the offspring and further work to directly assess mitochondrial respiration and thermogenic activation at various time points throughout the life course will be necessary to understand this observation with more clarity.

Contrary to HF/C mice our results showed that if mice born to HF dams were weaned onto an obesogenic diet, these mice displayed increased body weight, adiposity and serum cholesterol levels as well as glucose intolerance. These mice also exhibited slightly decreased RER and energy expenditure compared C/C mice. Data on RER, although limited due to low sample size and minimal effect, should be followed on to assess the metabolic repercussions of this finding. Increased body weight was comparable between HF/HF and C/HF, similar to a previous report by Loche et al.^22^. The effects of maternal and offspring high-fat diet were suggestive of an additive effect on serum triglycerides and FFAs levels. This is in agreement with previous reports describing a consistent^23^ or a trend^18^ towards increased serum levels of triglycerides in similar murine models, albeit using younger mice. Increased triglyceredaemia has been shown to aggravate other metabolic functions, such as insulin resistance (for review see^24^). Moreover, hypertriglyceridaemia was correlated with increased platelet activity both after acute intralipid injection^25^ and in chronic obese patients^26^. In spite of these previous reports, we show no correlation between platelet reactivity and circulating levels of HDL, LDL, triglycerides or FFAs (Supplementary Fig. S4), suggesting circulating lipids are unlikely to be involved in the increased platelet activation in HF/HF offspring.

Maternal or offspring high-fat diet ingestion had an overall effect of increasing MPV in offspring with no significant changes to platelet count. Of note, female offspring did not present increased platelet function despite severe metabolic dysfunction (Supplementary Fig. S5), suggesting a sex-specific effect. Although there are limited studies focusing on how female offspring respond to cardiometabolic insults, we suggest female offspring show a milder phenotype than males. However, further work is required to characterize how maternal obesity may influence platelets of female offspring.

In males, platelets from HF/HF mice had increased spreading over collagen, and presented increased fibrinogen binding when activated with ADP or CRP. This effect was not observed when thrombin was used, reiterating that increased platelet function in HF/HF is not fully explained by increased MPV alone. Higher MPV and platelet hyperactivation have been shown to be associated with increased platelet turnover in chronic diseases^27^. It is possible that platelets from HF/HF mice were younger, therefore with increased size and reactivity. Indeed, Grove et al. have shown that number of immature platelets was correlated with increased platelet aggregation^28^. Altogether, these findings suggest that HF/HF mice had hyperactive platelets, possibly due to alterations in platelet generation or higher platelet turnover. Future work is planned to assess proteins relevant to CRP and ADP signalling that may be involved in the phenotype herein described.

The fact that only HF/HF mice presented increased platelet activation suggests a previously undescribed ‘double-hit’ effect of maternal and offspring obesity, in which both insults are needed to alter platelet function. This is in line with a previous epidemiological report describing an association between maternal obesity and premature mortality from cardiovascular events^29^, to which platelets are intrinsically related^30^. Therefore, it is possible that the platelet hyperactivation herein observed in HF offspring born to HF dams can be a key pathophysiological component of the cardiovascular consequences of maternal obesity described in humans. Furthermore, Sousa et al.^31^ have shown that high-sucrose-fed mice that were subsequently fed a high-protein or normal diet for 12 weeks recovered some metabolic function (reduced fasting glycaemia, cholesterol, improved peripheral insulin sensitivity, etc.). Thus, it is possible that platelet hyperactivity can be recovered in offspring following nutritional interventions. Future research will explore epigenetic changes in platelets and megakaryocytes to further assess the potential ‘double-hit’ effect of maternal and offspring HF diet ingestion to better understand these and other possibilities.

Platelet GPVI and integrin α2 expression were decreased due to maternal high-fat diet ingestion. These alterations could be a consequence of shedding, in case of GPVI^32^ or epigenetic changes in case of integrin α2. Considering that HF/HF mice presented increased levels of ROS in platelets and that GPVI activation is both cause and consequence of increased ROS production in platelets^33^, it is possible that oxidative stress interferes with GPVI expression and response to CRP and collagen. However, increased platelet reactivity of HF/HF mice is unlikely to be solely due to alterations within the GPVI pathway, since these platelets were hyperactive when stimulated with ADP and it has been shown that GPVI-specific signalling (e.g. when using CRP) is not dependent on secondary mediators^34^. Therefore, there may be developmental programming at the epigenetic level based on the metabolic profile of the mother that affects several pathways that culminate in increased platelet function.

Barrachina et al. have recently shown that platelets from obese individuals are hyperresponsive to CRP and that they express higher levels of GPVI when compared to non-obese individuals^35^. Using non-human primates, Arthur et al.^36^ demonstrated that platelets from diabetic monkeys produce more ROS and are more responsive to CRP despite having unaltered levels of GPVI. There is a lack of reports assessing platelet GPVI levels in obesity and, although not directly comparable, the abovementioned studies flag the importance of the GPVI signalling pathway to the platelet dysfunction observed metabolic diseases. We argue that this might also be true for the consequences of maternal obesity on the platelet hyperactivation seen in obese offspring.

We acknowledge several limitations in this study. As this is the first report on the effect of maternal obesity on platelet function, we did not exhaust all aspects of platelet function. Future research could use different approaches, such as: platelet aggregation, calcium mobilization, and thrombus formation in vitro. Likewise, epigenetic changes due to maternal obesity were not explored and could provide interesting insights on the precise mechanism of the phenotype herein observed. We recognize that data in vitro do not always translate in functional consequences in vivo and therefore encourage future reports to assess the effects of maternal obesity on thrombus formation in vivo and link animal data with human studies to support or discard our hypothesis. There were reduced sample sizes for some experiments, such as measurement of ROS, which limits the interpretation of these data. Finally, we believe that future studies should address sex-specific effects of maternal metabolic dysfunction on platelet function.

To summarize, we propose that platelets can be programmed by metabolic dysfunction in mothers and that there is a ‘double-hit’ effect that leads to platelet hyperactivity. The molecular mechanisms involved decreased GPVI expression and increased ROS production. Also, maternal high-fat diet ingestion per se seemed to induce a pro-adaptive metabolic response in standard laboratory diet-fed offspring, since these animals were leaner and tended to burn more fat than their counterparts born to lean dams. Future studies should address the consequences of platelet hyperactivation in the development of cardiac events in offspring born to obese dams. Altogether, these findings shed light on possible pathophysiological explanations to the increased risk of cardiovascular events in individuals born to mothers with metabolic dysfunction and add yet another layer of evidence to the deleterious effects of maternal obesity to the health of their offspring.

## Methods

The data that support the findings of this study are available from the corresponding author upon reasonable request.

### Reagents

Prostacyclin (PGI_2_), Adenosine Diphosphate (ADP), thrombin, Gly-pro-arg-pro pep-tide (GPRP), 2′,7′-Dichlorofluorescin diacetate (DCFDA) and dihydroethidium (DHE) were purchased from Sigma-Aldrich (Dorset, UK). FITC-conjugated anti-fibrinogen was purchased from Agilent (Stockport, UK). Collagen was purchased from Nycomed (Munich, Germany) and Collagen-Related Peptide (CRP) was obtained from Prof Richard Farndale (University of Cambridge, Cambridge, UK). Alexa-488 conjugated phalloidin was purchased from Life Technologies (Paisley, UK). Rat anti-mouse GPVI, α2 integrin, GpIbα and appropriate IgG controls were purchased from Emfret (Emfret Analytics GmbH & Co, Eibelstadt, Germany). Goat anti-mouse CD36 was purchased from R&D Systems (R&D Systems Inc, Abingdon, UK).

### Animal and experimental design

All animal studies were approved by the Medical Research Council Harwell Institute Animal Welfare and Ethical Review Board, and all procedures were carried out within project license restrictions (PPL 30/3146) under the UK Animals (Scientific Procedures) Act 1986 and conform to the guidelines from Directive 2010/63/EU of the European Parliament on the protection of animals used for scientific purposes. Female C57BL6/N mice at 8 weeks old were fed either a control diet (C, 10% kCal fat) or a high-fat diet (HF, 60% kCal fat) for 6 weeks before pregnancy. They were then mated at 14 weeks of age with standard laboratory diet-fed males and maintained on their respective diets during pregnancy (3 weeks) and lactation (4 weeks), totalling 13 weeks of dietary intervention on the dams, as described previously^37^. Upon weaning at 4 weeks, offspring were randomly assigned by a technician, a control or high-fat diet for 26 weeks. Therefore, there were 4 experimental groups: C-fed dam and C-fed offspring (C/C), C-fed dam and HF-fed offspring (C/HF), HF/C and HF/HF. For the male offspring study, the two maternal dietary regimes generated 11 Control diet (C) litters and 11 high-fat diet (HF) litters which were randomly allocated to the two offspring dietary groups: offspring from C-dams to C–C or C-HF and offspring from HF-dams to HF-C or HF-HF. The new groups are composed from different litters, some originate from HF litters, others from C litters. Out of these, littermates (often more than 1) were randomized across different C or HF groups to avoid littermate bias (one litter was often used in more than one group), therefore, out of the 11 litters for C dams and 11 litters for HF dams, there were: 7 litters for HF/HF group, 9 litters for HF/C group, 6 litters for C/HF group and 7 litters for C/C group. The specific number of litters per group per experiment is presented in figure legends. For the female offspring study, there were 11 litters from C dams and 12 litters from HF dams, which were randomized in: 10 litters for HF/HF group, 10 litters for HF/C group, 8 litters for C/HF group and 9 litters for C/C group. Dams were analysed before mating to confirm obesity induction (Supplementary Fig. S1). Throughout dietary intervention, mice were weighed weekly, metabolic cage analysis performed at 22 weeks whilst body composition and intraperitoneal glucose tolerance test (ipGTT) were measured at 26 weeks of age. At 30 weeks of age, blood was collected through retro-orbital sinus with mice under terminal anaesthesia, which consisted of inhalation of 5% isoflurane until full anaesthesia was achieved, determined by loss of pedal reflex. Death was confirmed by cessation of the circulation and neck dislocation. Interscapular brown adipose tissue (BAT) was isolated, collected and kept in Invitrogen TRI Reagent for RNA extraction. For serological analysis, blood was allowed to clot, then centrifuged at 3000×*g* for 3 min and sera frozen at -80 °C until analysis. For platelet studies, blood was collected in 1:9 v/v acid citrate dextrose (ACD: 2.5% sodium citrate, 2% D-glucose, and 1.5% citric acid) and analysed after 1 h of resting. A schematic diagram of the animal model can be found in Supplementary Fig. S2.

### Serological analyses and full blood count

All samples for serological analyses and full blood count were processed by the MRC Clinical Chemistry core facility, MRC Harwell Institute (Oxfordshire, UK) using an AU680 Clinical Chemistry Analyser (Beckman Coulter, High Wycombe, UK) and performed as per manufacturer (Addenbrokes Hospital, Cambridge, UK). Full blood count was determined using mice-specific settings on an Advia 2120 dedicated veterinary blood counter (Siemens Healthcare, Surrey, UK). TyG index was calculated as: Ln [triglyceridaemia (mg/dL) x glycaemia (mg/dL)]/2.

### Body composition analysis

Body composition was measured at 26 weeks of age by nuclear magnetic resonance (EchoMRI, Houston, Texas, USA), which determined total body fat, lean mass and free water in grams. The percentage of each component was then calculated based on the total body weight of the animal.

### Intraperitoneal glucose tolerance test

Intraperitoneal glucose tolerance tests were performed at 26 weeks following a 16 h fast. Specifically, fasted mice received an i.p. administration of 2 g/kg glucose and blood sampled under a local anesthetic at 0 min (baseline), 15, 30, 60 and 120 min post glucose injection. Whole blood glucose was measured using an AlphaTRAK meter and test strips (Abbott Animal Health, UK).

### Indirect calorimetry

Indirect calorimetry was performed in 22 weeks old mice using a TSE PhenoMaster system (TSE Systems GmbH, Hamburg, Germany). Animals were individually placed in metabolic cages for 1 h to acclimatize. Measurements started at 15:00 and finished at 11:00 of the next day. VO_2_, VCO_2_, respiratory exchange rate (RER), energy expenditure rate and locomotor activity were measured every 15 min under constant temperature of 20 °C.

### RNA isolation, cDNA synthesis and qRT-PCR

Levels of mRNA were measured exactly as described previously^38^. Briefly, interscapular BAT was isolated in Invitrogen TRI Reagent, lysed and homogenized with QuickPrep Adaptor (Fisher Scientific). RNA isolation was performed according to the manufacturer (Fisher Scientific) and cDNA synthesized using the Applied Biosystems High-Capacity cDNA Reverse Transcription Kit (Fisher Scientific), Invitrogen RNaseOUT Recombinant Ribonuclease Inhibitor (Fisher Scientific) and T100 Thermal cycler (Bio-Rad). Gene expression of cDNA (30 ng) was determined using the qPCRBIO Probe Mix No-ROX (PCR Biosystems), performed on the MyiQ Single-Colour Real-Time PCR Detection System (Bio-Rad). Samples were measured in duplicates and the 2^−∆∆^Ct method employed to determine fold change in gene expression. The target genes were normalized to the housekeeping gene, peptidylprolyl isomerase A (PPIA). All Applied Biosystems TaqMan Gene Expression Assay (FAM-MGB) (Major Resources Table) were purchased from Fisher Scientific.

### Platelet activation and membrane receptor studies

Blood was collected through retro-orbital sinus in 1:9 v/v ACD tubes and centrifuged at 203×*g* for 8 min to separate the platelet-rich plasma (PRP). PRP was then incubated in a 96-well plate with or without NO donor PAPA-NONOate at 100 µM for 10 min prior to addition of agonists ADP, CRP or thrombin at specified concentrations. In experiments containing thrombin, 0.5 mg/mL GPRP was added to prevent fibrin polymerization. FITC-labelled fibrinogen was incubated for 30 min in the dark followed by dilution (25X) with Tyrodes-HEPES buffer (134 mM NaCl, 20 mM N-2-hydroxyethylpiperazine-N′-2-ethanesulfonic acid, 12 mM NaHCO3 5 mM glucose, 0.34 mM Na_2_HPO_4_, 2.9 mM KCl and 1 mM MgCl_2_, pH 7.3). In order to measure platelet membrane receptor expression, whole blood was incubated with antibodies specific to membrane receptors or control IgG at the concentrations specified by the manufacturer for 30 min in the dark. In both assays, events were acquired using a BD Accuri C6 Plus flow cytometer (BD Biosciences, Oxford, UK) and platelets gated by forward and side scatter.

### Platelet spreading

PRP was supplemented with 1.25 µg/mL PGI_2_ and further centrifuged at 1028 × *g*or 5 min and washed platelets (WP) pellet resuspended in Tyrodes-HEPES buffer. WP (2 × 10^7^ platelets/mL) were added to a coverslip coated with 100 µg/mL collagen and left to adhere for 45 min at 37 °C. Non-adherent platelets were washed off with PBS, and adherent platelets fixed with 0.2% paraformaldehyde, permeabilized with Triton-X 0.01% v/v for 10 min and subsequently stained with Alexa Fluor 488-conjugated phalloidin (1:1000 v/v) for 1 h in the dark at room temperature. Images were acquired using a Nikon A1-R Confocal microscope (Nikon Instruments Europe BV, Amsterdam, Netherlands).

### Reactive oxygen species (ROS) measurement

Whole blood (WB) was incubated with 20 µM DCFDA for 30 min or 10 µM DHE for 15 min in the dark and events acquired using a BD Accuri C6 Plus flow cytometer. Platelet and red blood cells populations were separated by forward and side scatter.

### Statistical analysis

Statistical analyses were run in GraphPad Prism 8.0 software (GraphPad Software, San Diego, USA). Quantitative results in figures and tables were expressed as mean ± SEM and individual values. Overall, n = 17 for C/C, n = 10 for C/HF, n = 16 for HF/C and n = 13 for HF/HF. However, n number varied across assays. For metabolic and phenotypic studies n = 10–17 per group except for indirect calorimetry experiments, which had n = 3–8 due to low availability of metabolic cages and time-constraints. Functional platelet studies had n = 5–13. All groups in all experiments were derived from at least 3 different litters. Outliers were identified and excluded using ROUT method. Data were considered of normal distribution due to sample size, as recommended previously^39^. Equal variance (sphericity) was not assumed since samples were independent and analysed through unpaired two-way ANOVA for bar graphs with Tukey’s post-test. The two-way ANOVA models were deemed additive if no interaction was detected, as described previously^22,40^. Simple linear regressions were calculated between circulating lipids and platelet reactivity to test for linear correlations. Overall effect of maternal, offspring or the interaction of these are presented and interpreted throughout text. Data in XY graphs were assessed by repeated measures two-way ANOVA and Tukey’s as post-test with level of significance of 5%.

## Supplementary Information

Supplementary Information 1.

Supplementary Information 2.

## Abbreviations

ADRβ3: Adrenergic receptor beta 3
COX7A1: Cytochrome c oxidase subunit 7A1
COX8B: Cytochrome c oxidase subunit 8B
CRP: Collagen-related peptide
DCFDA: 2′,7′-Dichlorofluorescin diacetate
DHE: Dihydroethidium
DIO2: Iodothyronine deiodinase 2
FFAs: Free fatty acids
GPVI: Glycoprotein VI
ipGTT: Intraperitoneal glucose tolerance test
NO: Nitric Oxide
PGC1α: Peroxisome proliferator-activated receptor gamma coactivator 1-alpha
PRP: Platelet-rich plasma
RER: Respiratory exchange rate
ROS: Reactive oxygen species
UCP1: Uncoupling protein 1
WB: Whole blood
WP: Washed platelets
