# Supplementary material for: Maternal and offspring high-fat diet leads to platelet hyperactivation in male mice offspring
Source: Sci Rep. 2021 Jan 14;11:1473. doi: 10.1038/s41598-020-80373-3 (PMC7809045; doi:10.1038/s41598-020-80373-3)
Supplement: Supplementary file 1 — Supplementary Information 1. [file 41598_2020_80373_MOESM1_ESM.pdf]

Maternal and offspring high-fat diet leads to platelet hyperactivation in male mice offspring

Running title: Maternal high-fat diet alters platelet function in obese offspring

Authorship

Renato S. Gaspar [a*], Amanda J. Unsworth [b], Alaa Al-Dibouni [a], Alex Bye [a], Tanya Sage [a], Michelle Stewart [c], Sara Wells [c], Roger D. Cox [d], Jonathan M. Gibbins [a], Dyan Sellayah [a#], Craig Hughes [a#]

Affiliations

[a] Institute for Cardiovascular and Metabolic Research, School of Biological Sciences, University of Reading, Reading, UK.

[b] Department of Life Sciences, Faculty of Science and Engineering, John Dalton Building, Manchester Metropolitan University, Manchester, UK, M1 5GD

[c] MRC Harwell Institute, Mary Lyon Centre, Harwell Campus, Oxfordshire, OX11 0RD, UK.

[d] MRC Harwell Institute, Genetics of type 2 diabetes, Mammalian Genetics Unit, Harwell Campus, Oxfordshire, OX11 0RD, UK.

# Joint senior authors

*** Corresponding author**

Renato Simões Gaspar, M.D.

Institute of Cardiovascular and Metabolic Research, School of Biological Sciences. University of Reading - Harborne Building, Reading, RG6 6AS, UK.

E-mail: renatosgaspar@gmail.com, phone: +44 11 8378 7047

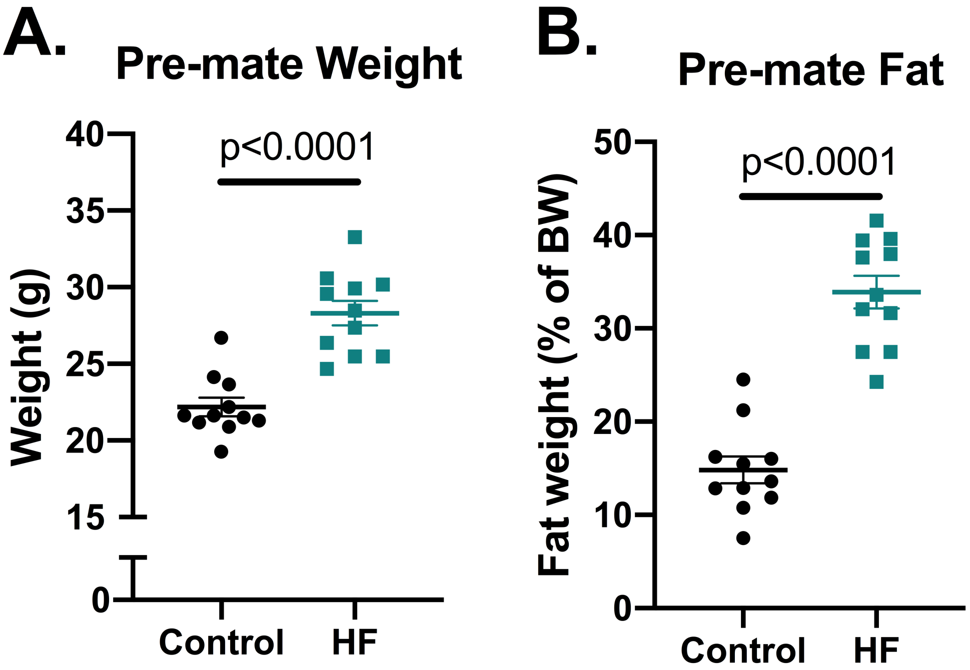

Supplementary Figure 1. Increased body weight and fat accumulation in HF dams before mating. 3 weeks old female C57BL6/N mice were fed a control standard laboratory diet (C) or high-fat (HF) diet for 6 weeks before pregnancy. Body weight and fat pads measured using nuclear magnetic resonance (EchoMRI™, Houston, Texas, USA). n=11 for each group. Data presented as mean ± SEM and analysed through unpaired Student t-test.

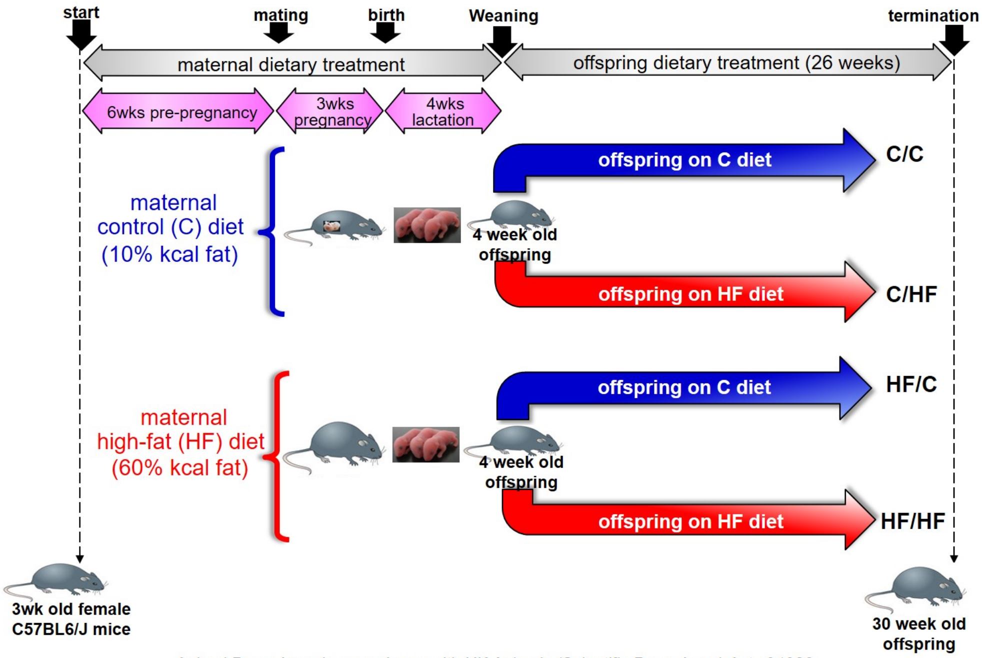

Animal Procedures in accordance with UK Animals (Scientific Procedures) Act of 1986

Supplementary Figure 2. Design of animal experiments. 3 weeks old female C57BL6/N mice were fed a control standard laboratory diet (C) or high-fat (HF) diet for 6 weeks before pregnancy, as well as the whole duration of pregnancy and lactation. Offspring was then subdivided to receive C or HF for 26 weeks after weaning, constituting four experimental groups: C/C, dam and offspring fed standard laboratory diet; C/HF dam fed standard laboratory diet and offspring fed high-fat diet; HF/C dam fed high-fat diet and offspring fed standard laboratory diet; HF/HF dam and offspring fed high-fat diet. Offspring was terminated at 30 weeks of age.

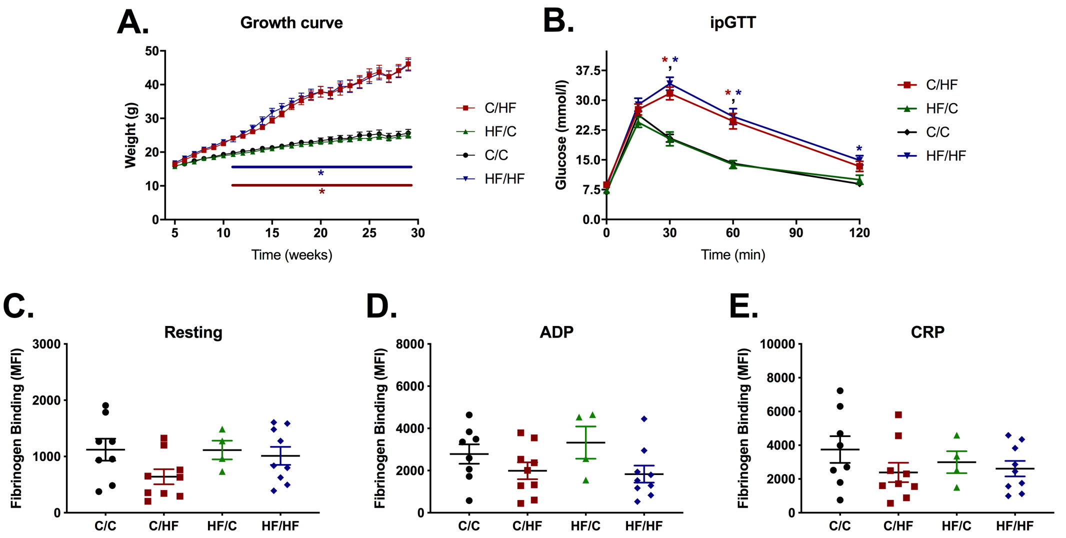

Supplementary Figure 3. Growth curve, ipGTT and platelet reactivity in female offspring. (A) Growth curve with weekly weight measures. (B) Intraperitoneal glucose tolerance test (ipGTT) with glucose measured at 0, 15, 30, 60 and 120 min after glucose injection. Fibrinogen binding measured in whole blood at resting (C), stimulated with 10 µM ADP (D) or 3 µg/mL CRP (E). N=4-9 mice per group. Graphs show mean ± SEM and individual values. P-value is presented within each graph. *$p<0.05$ and colour of stars indicate significance of the group against C/C.

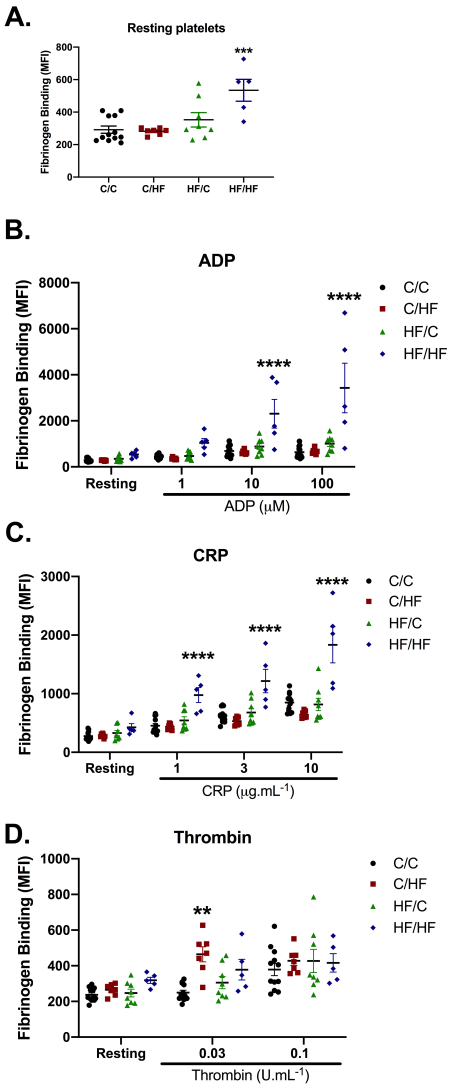

Supplementary Figure 4. Platelet hyperactivation of HF/HF mice is maintained throughout different doses of ADP and CRP, but not thrombin. Platelet-rich plasma (PRP) was incubated with FITC-conjugated fibrinogen and binding was measured in resting (A) or ADP (B), CRP (C) or Thrombin (D) stimulated platelets. C/C, dam and offspring fed standard laboratory diet; C/HF dam fed standard laboratory diet and offspring fed high-fat diet; HF/C dam fed high-fat diet and offspring fed standard laboratory diet; HF/HF dam and offspring fed high-fat diet. N=5-13 mice per group. Graphs show mean ± SEM as well as individual values. ** $p<0.01$ *vs* C/C; *** $p<0.001$ *vs* C/C; **** $p<0.0001$ *vs* C/C. Data analysed by repeated-measures two-way ANOVA with Tukey's multiple comparisons test. The overall effects of maternal and offspring diet are reported where significant.

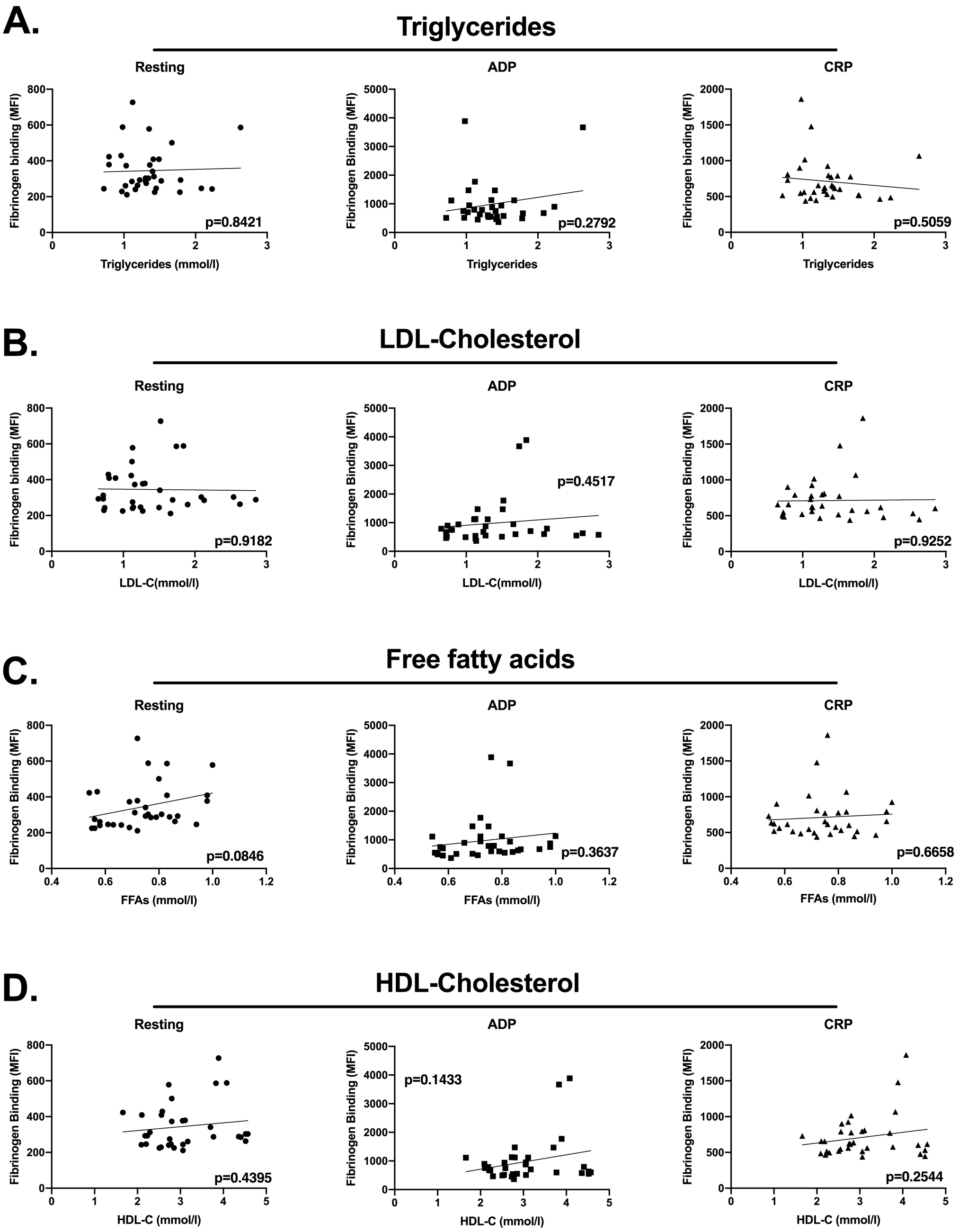

Supplementary Figure 5. Correlation between circulating lipids and platelet reactivity. Circulating serum lipids were measured using an AU680 Clinical Chemistry Analyser (Beckman Coulter, High Wycombe, UK). Fibrinogen binding was measured using a flow cytometer in platelet-rich plasma (PRP) stimulated with ADP, CRP or kept at resting state. Linear regression was performed to determine if there was a correlation. N=5-13 mice per group. Graphs show individual values and line of linear regression. P-value is presented within each graph.

Major Resources Table

In order to allow validation and replication of experiments, all essential research materials listed in the Methods should be included in the Major Resources Table below. Authors are encouraged to use public repositories for protocols, data, code, and other materials and provide persistent identifiers and/or links to repositories when available. Authors may add or delete rows as needed.

Animals (in vivo studies)

**Species**	**Vendor or Source**	**Background Strain**	**Sex**	**Persistent ID / URL**
M.musculus	In house	C57BL6/N	F/M	

Genetically Modified Animals

Not applicable

Antibodies

**Target antigen**	**Vendor or Source**	**Catalog #**	**Working concentration**	**Lot # (preferred but not required)**	**Persistent ID / URL**
F-actin	Life Technology	#8878	1:1000 v/v		https://www.cellsignal.com/products/buffers-dyes/alexa-fluor-488-phalloidin/8878
GPVI	Emfret	M011-1	5 µL per 10^6 platelets		https://www.emfret.com/uploads/tx_beproducts/M011-1_JAQ1.pdf
α2 integrin	Emfret	M070-1	5 µL per 10^6 platelets		https://www.emfret.com/uploads/tx_beproducts/M070-1_SamG4.pdf
GpIbα	Emfret	M040-2	5 µL per 10^6 platelets		https://www.emfret.com/uploads/tx_beproducts/M040-2_XiaG5.pdf
Negative control (Rat IgG)	Emfret	P190-2	5 µL per 10^6 platelets		https://www.emfret.com/uploads/tx_beproducts/P190-2_rat_IgG.pdf
Negative control (Rat IgG)	Emfret	P190-1	5 µL per 10^6 platelets		https://www.emfret.com/uploads/tx_beproducts/P190-1_rat_IgG.pdf
CD36	R&D Systems	AF2519	2.5 µg per 10^6 platelets		https://www.rndsystems.com/products/mouse-cd36-sr-b3-antibody_af2519
Tyrosine phosphorylation (4G10 antibody)	Merck Millipore	05-321	1:1000 v/v		https://www.merckmillipore.com/GB/en/product/Anti-Phosphotyrosine-Antibody-clone-4G10,MM_NF-05-321?ReferrerURL=https%3A%2F%2Fwww.google.com%2F
Actin	Abcam	ab8229	1:1000 v/v		https://www.abcam.com/beta-actin-antibody-loading-control-

					ab8229.html
$VASP_{S239}$	Cell Signaling	#3114	1:1000 v/v		https://www.cellsignal.com/products/primary-antibodies/phospho-vasp-ser239-antibody/3114
Akt_{S473}	Cell Signaling	#9271	1:1000 v/v		https://www.cellsignal.com/products/primary-antibodies/phospho-akt-ser473-antibody/9271?site-search-type=Products

DNA/cDNA Clones

Clone Name	Sequence	Source / Repository	Persistent ID / URL
ADRβ3	Mm02601819_g1	Fisher Scientific	https://www.thermofisher.com/order/genome-database/details/gene-expression/Mm02601819_g1
COX7A1	Mm00438297_g1	Fisher Scientific	https://www.thermofisher.com/order/genome-database/details/gene-expression/Mm00438297_g1
COX8B	Mm00432648_m1	Fisher Scientific	https://www.thermofisher.com/order/genome-database/details/gene-expression/Mm00432648_m1
DIO2	Mm00515664_m1	Fisher Scientific	https://www.thermofisher.com/order/genome-database/details/gene-expression/Mm00515664_m1
PGC1α	Mm01208835_m1	Fisher Scientific	https://www.thermofisher.com/order/genome-database/details/gene-expression/Mm01208835_m1
UCP1	Mm01244861_m1	Fisher Scientific	https://www.thermofisher.com/order/genome-database/details/gene-expression/Mm01244861_m1

Cultured Cells
Not applicable.

Data & Code Availability
Not applicable.

Other
Not applicable.
