# Supplementary figures and images for: Maternal and offspring high-fat diet leads to platelet hyperactivation in male mice offspring
Source: Sci Rep. 2021 Jan 14;11:1473. doi: 10.1038/s41598-020-80373-3 (PMC7809045; doi:10.1038/s41598-020-80373-3)

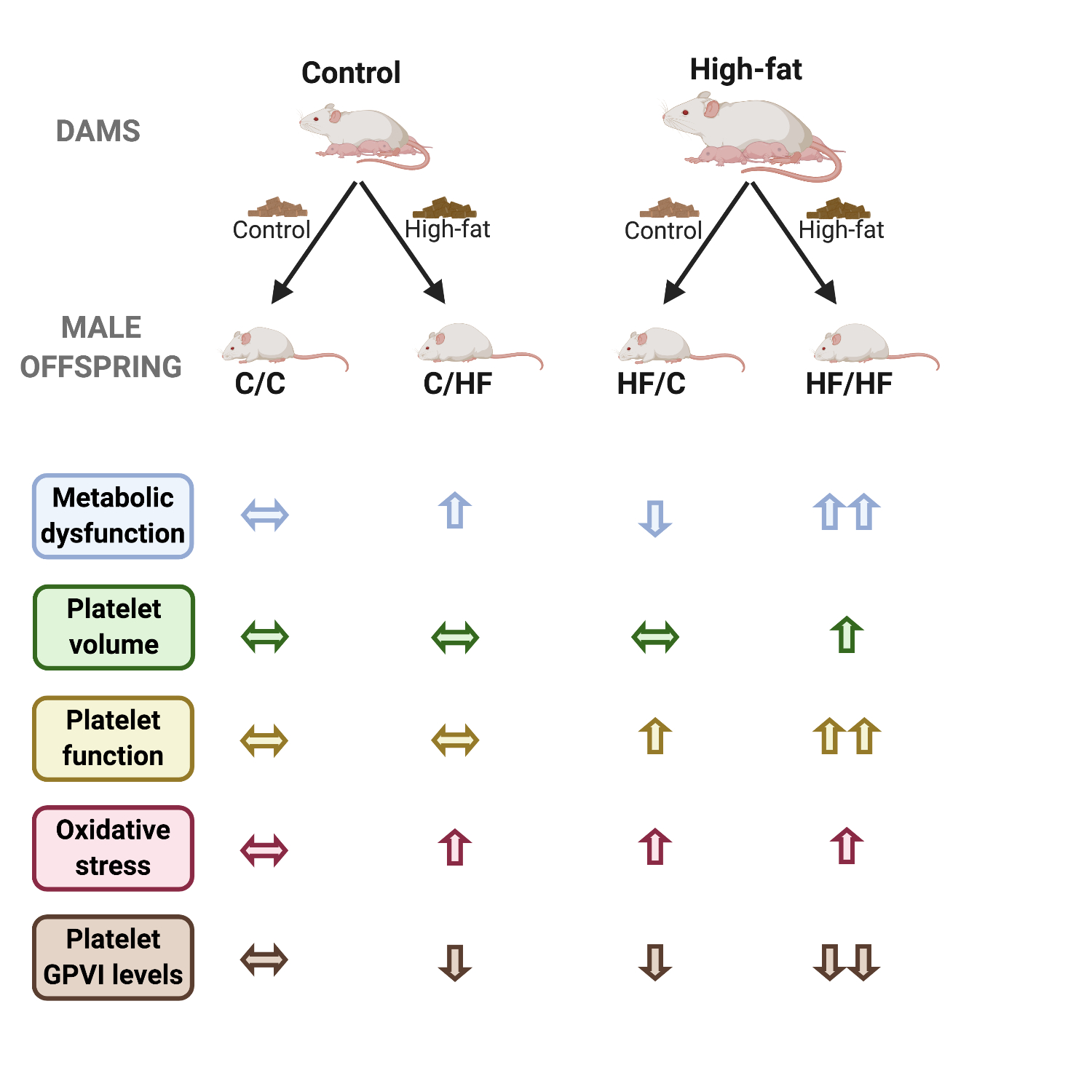

Supplement: Supplementary file 2 — Supplementary Information 2. [file 41598_2020_80373_MOESM2_ESM.jpeg]
